# The novel circ_0028171/miR-218-5p/IKBKB axis promotes osteosarcoma cancer progression

**DOI:** 10.1186/s12935-020-01562-8

**Published:** 2020-10-06

**Authors:** Feng Pan, Jun Zhang, Benseng Tang, Li Jing, Bing Qiu, Zhengang Zha

**Affiliations:** 1 Department of Bone and Joint Surgery, Institute of Orthopedic Diseases, The First Affiliated Hospital, Jinan University, Guangzhou, Guangdong China; 2 Department of Bone and Joint Surgery, Gui Zhou Orthopedic Hospital, Gui Zhou, China

**Keywords:** Circ_0028171, miR-218-5p, IKBKB, osteosarcoma, growth, metastasis

## Abstract

**Background:**

Recently, it has been demonstrated that circular RNA (circRNA) contributes to the production and progression in human cancer. However, the specific function and underlying mechanism of circ_0028171 in osteosarcoma (OS) still remain largely unclear and require to be investigated.

**Methods:**

In our study, we confirmed differentially expressed circRNAs by microarray analysis in normal bone cells vs. OS cell lines. The expression of circ-0028171 in OS was measured by qRT-PCR. Nuclear-cytoplasmic fractionation was employed to identify the localization of circ-0028171, and RNase R and actinomycin D treatment were used to prove its circular characteristic. In vitro experiments, such as CCK-8 method, cell count, cell colony formation, transwell migration and invasion assays, and in vivo tumor models were adopted to evaluate the effect of circ_0028171. Further, luciferase reporter, RIP and RNA pull-down assays were conducted to confirm the binding sites of circ_0028171 with miR-218-5p.

**Results:**

We found that circ_0028171 displayed a remarkably higher expression in both OS tissues and cell lines. Circ_0028171 mainly located in the cytoplasm as a stable cyclic transcript. Knockdown of circ_0028171 suppressed OS tumor growth in vitro and in vivo, while up-regulated circ_0028171 remarkably enhanced cell proliferation, migration and invasion abilities in OS. Several mechanistic experiments revealed that circ_0028171 served as a sponge of miR-218-5p to increase IKBKB expression.

**Conclusions:**

our research reveals that circ_0028171 might promote the malignant behavior of OS tissues through miR-218-5p/IKBKB axis, which could be a potential novel marker for early diagnosis of OS.

## Background

Osteosarcoma (OS) is the third prevalent and lethal malignant bone tumor among children and adolescents. A great number of cancer-related deaths are caused by Osteosarcoma annually due to its fast proliferation, high metastatic properties, and chemo-resistance [1, 2]. In recent years, a wide variety of medical therapies have been proposed for Osteosarcoma, including surgery, chemotherapy, radiotherapy, hormonotherapy, and small molecular targeting treatments. Indeed, we have obtained promising results, as previously reported, the prognosis of OS patients without metastasis is generally excellent, and the five-year survival is greater than 60% [3]. Nevertheless, the prognosis of patients with advanced disease is very poor because of the recurrence and distant metastasis [4]. Therefore, more practical researches and investigations were needed to discover highly sensitive and specific markers for the early diagnosis and explore more effective therapeutic strategies.

In recent years, researches [5–7] on circRNAs has become a burgeoning topic in the domain of malignant tumors. CircRNAs [6], a new-found type of special endogenous noncoding RNAs, is characterized by lack of a 5′-end cap and a 3′-end poly(A) tail and forming covalently-closed continuous loops. CircRNAs were grouped into four categorized [7] due to there structure. Moreover, circRNAs [8] were discovered in various eukaryotic organisms from protists to humans and accumulating evidence [9] showed that circRNAs were abnormally expressed in multiple types of malignant tumors and these dysregulated circRNAs contributed to the diagnosis and prognosis of patients, such as thyroid cancer, epithelial ovarian cancer, hepatocellular carcinoma, cervical cancer and colorectal cancer [10–14]. Substantial evidence [15, 16] has revealed that circRNAs play vital roles in regulating potential biological functions of cancers by acting as miRNA sponge ; regulating gene splicing, transcription and translation; serving on autophagy regulator and interacting with RNA-Binding proteins (RBPs). In OS, several circRNAs have been identified to participate in cell proliferation, migration, apoptosis, and drug resistance [17]. However, the function and mechanism of circ_0028171, a newly discovered circRNA, need to be further explored in OS.

As one type of significant functional small non-coding RNAs, microRNAs (miRNAs) [18] has been studied widely. A large number of studies [19–21] have confirmed that microRNAs is extensively involved in regulation of multiple biological functions, and regulates cell cycle, cell proliferation, differentiation, apoptosis. Regarding miR-218, Yao et al. [22] reported that microRNA-218 inhibited the viability and accelerates the apoptosis in ARPE-19 cells by directly targeting RUNX2. Moreover, the experimental research showed [23] that miR-218 was markedly downregulated in diabetic nephropathy rat model, and overexpressed miR-218 markedly reduced inflammatory responses, detected by measuring the level of TNF-α, IL-6, IL-1β, and MCP-1. Previously, it [24] has been reported that lncRNA CCAT1 serves as a microRNA-218 sponge to induce Gefitinib Resistance in NSCLC by Targeting HOXA1. In fact, Previous studies [25, 26] had identified that miR-218 exhibited low expression and inhibited tumor growth in osteosarcoma. However, the upstream regulating mechanism and downstream targets of miR-218 still need to be explored.

It [27] is well known that IKK complex consists of three subunits, including IKKα, IKKβ(also called IKBKB), and IKKγ. In addition, IKBKB [28, 29] has been shown to take part in tumor growth via NF-κB activation and the phosphorylation-dependent inhibition of tumor suppressors. A previous study [30] indicated that the protein expression levels of IKBKB were remarkably decreased in U-2OS cells after treated with Chanti-TRIM, while TRIM induced the expression levels of p65, IKKβ and IκBα. The above finding suggested that Chanti-TRIM may impede the aggressive phenotypes via the MMP-9-induced NF-κB signaling pathway in vitro. Hu et al. [29] concluded that IKBKB could phosphorylate FOXO3a, a tumor-suppressive forkhead transcription factor, leading to increased cell proliferation, cell cycle progression and tumorigenesis. Nonetheless, little was known about the fuction of IKBKB in osteosarcoma.

In our study, we discovered that the expression level of circ_0028171 was remarkably higher in OS tissues than in surrounding normal tissues. Furthermore, our data showed that circ_0028171 facilitated cell proliferation, migration, invasion through miR-218-5p/IKBKB axis in OS.

## Materials and methods

### Patient selection and tissue specimens

20 matched pairs of fresh frozen OS tissue samples and surrounding normal tissues were collected from OS patients at The First Affiliated Hospital of Jinan University between April 2017 and April 2019. All patients had signed the informed consents. Our project was approved by the Ethics Committee of. Tissue samples were preserved in liquid nitrogen until use.

### Cell culture

Human Osteoblasts cell (HOB1) and five human osteosarcoma cell lines (HOB1, MG63, Saos-2, U2OS,143B) were purchased from ATCC, All the cell lines were cultured in RPMI-1640 medium (HyClone, Logan, UT, USA) with 10% fetal bovine serum (FBS, Gibco, Australia).

### Cell transfection

Small interference RNAs (siRNAs) specifically targeting to hsa-circ_0028171 and IKBKB, miR-218-5p mimic, inhibitor and their negative controls were generated by Gene-Pharma (Shanghai, China). TO induce the overexpression of circ_0028171 and IKBKB, KBKB,0028171verexprs were cloned into pEX-3 vector, respectively (Shanghai GenePharma CO. Ltd). All above vectors were transfected with Lipofectamine®ipofectInvitrogen, Thermo Fisher Scientifific, USA). After transfection for 48 h–72 h, transfection efficiency was measured by qRT-PCR.

### Quantitative real-time PCR

Total RNA was extracted by Trizol (Invitrogen, CA, USA). Then, 1 µl of RNA was used to synthesize the complementary DNA (cDNA) with the Transcriptor First Strand cDNA Synthesis Kit (Roche, Mannheim, Germany). qRT-PCR reactions were performed in Roche Real-Time PCR System in triplicates. GAPDH mRNA or U6 was served as a normalization. The primer sequences were shown in the Table 1.

**Table 1:** The primers used in the quantitative real-time PCR assay

Name	Sequence (5′ → 3′)
circRNA_0028171	
Forward	CAGGGCAGGGACAGGAAG
Reverse	GAGACAGGCAAGAGGACAAGG
GAPDH	
Forward	TGTGGGCATCAATGGATTTGG
Reverse	ACACCATGTATTCCGGGTCAAT
IKBKB	
Forward	GGAAGTACCTGAACCAGTTTGAG
Reverse	GCAGGACGATGTTTTCTGGCT
U6	
Forward	CTCGCTTCGGCAGCACA
RT	GTCGTATCCAGTGCAGGGTCCGAGGTGCACTGGATACGACAAAATATGGAAC
miR-218-5p	
Forward	GGGGGTTGTGCTTGATCTAAC
RT	GTCGTATCCAGTGCAGGGTCCGAGGTGCACTGGATACGACACATGGT
miR-1273e	
Forward	ACACTCCAGCTGGGTTGCTTGAACCCAGGA
RT	CTCAACTGGTGTCGTGGAGTCGGCAATTCAGTTGAGTCCACTTC
miR-583-5p	
Forward	GGTGCTCTATGGTAATCTAGCTG
RT	GTTCAGCCTAGTGCAGGGTCAGAGGTGCAGAGGAGACGACTTATACA
miR-3919	
Forward	GGTAGAGTTCTCTCAGTAGTAAC
RT	GTCGTATCCAGTGCAGGGTCCGAGGTGCACTGGATACGACAGGGATT
miR-6835-5p	
Forward	AGTCGAAGTCGAAGCTGTCATCG
RT	GGTCAGGTCAACTTGCACCTGCAAACTGTGGTAGGCTTAACCGTCATC
miR-6748-5p	
Forward	ATCGGATCAATGCTTAGCTAGAG
RT	GGCCTAAATGTAACATGCTAGCTAGTTGGATAAATGGTCCGATCGATC

### Analysis of circ-0028171 distribution

We used PARISed cytoplasmic fraction to isolated Nuclear and cytoplasmic RNA as previously described [11]. Then circ-0028171 expression level was detected in each fraction by qRT-PCR.

### Western blotting

After a 48-h transfection, we used RIPA lysis buffer with 1% protease inhibitor (Beyotime Biotechnology, China) to extract total protein. Then a BCA Protein Assay Kit (Beyotime Biotechnology, Jiangsu, China) was used to determine the concentrations of total cellular protein. The protein samples (40ug/lane) were loaded on to 10% SDS-PAGE gel and transferred onto PVDF membranes (Millipore, MA, USA) by electroblotting. After blocking for 1 h with 5% non-fat milk, the membranes were incubated overnight at 4 ℃ with primary antibodies. Next, the membranes were treated with secondary antibodies (Proteintech) for 1 h at room temperature. Finally, immunoblots results were visualized using ECL chemiluminescence detection system (Thermo Scientific). The primary antibodies were as follows: IKBKB (1:1000; Cell Signaling Technology, USA), GAPDH (1:6000, Sigma, St. Louis, MO, USA).

### RNase R and actinomycin D treatment

Actinomycin D and RNase were purchased from Sigma. For RNase R treatment, 10 µg of total RNA was incubated 30 min at 37 °C with or without 40 U of RNase R. Actinomycin D (10 µg/ml) was used to block transcription and RNAs were harvested at various time points after actinomycin D treatment. Then we detect the expression of ATP2A2 and circ_0028171 after treatment with RNase R or actinomycin D by qRT-PCR.

### Cell proliferation assay

The MG-63 and 143B cells were seeded into 96-well plates in quadruplicate with serum-free RPMI 1640 medium then cultured in a humidifified atmosphere with 5% CO_2_. Subsequencely, cell proliferation was measured at 24, 48 and 72 h. Before measurement, each well was added with 10 µl CCK-8 reagent (Beyotime Biotechnology, Jiangsu, China). Cell numbers in each well were also quantified by using Celigo image cytometer (Brooks Life Science Systems).

As for Colony formation assay MG-63 and 143B cells (0.5 × 103 per well) were seeded in six-well plate containing 2 ml growth medium with 10% FBS and maintained in a 37 °C, 5% CO_2_ incubator for 14 day. Finally, removed the medium, and the colonies (> 50 cells) were counted after staining with 0.1% crystal violet.

### Transwell migration and invasion assays

Regarding to cell migration assay, OS cells in serum-free medium were plated into 24-well plates and cultured in BD BioCoat Matrigel Invasion Chambers (8.0 um pore, BD company). Invasion assay was performed in the same way as the migration assay except that the membrane was coated with Matrigel (BD Biosciences, San Jose, CA, USA). Complete medium containing 20% FBS as a chemo-attractant was added into the lower chamber. After incubating for 12 h or 48 h, cells on the upper chamber were scraped off. Cells on the lower side were fixed using methanol and stained using 0.1% crystal violet solution, and were counted using Zeiss Photomicroscope dependent on at least five random fields.

### Luciferase reporter assay

For the luciferase reporter assay, MG-63 and 143B cells (5 × 10^3^) were seeded into 96-well plates and cultured until a confluence of 70% was reached. Subsequently, luciferase reporter plasmids were transfected into MG-63 and 143B cells using LipofectamineTM2000 reagent. After 48 h, the dual-luciferase reporter assay kit (Promega Corporation, Madison, WI, USA) was utilized for luciferase activity. All experiments were independently repeated in triplicate.

### RNA immunoprecipitation assay

RIP was performed using RIP RNA-Binding Protein Immunoprecipitation Kit (Millipore). Then purified RNA was used to detect miR-218-5p and circ_0028171 expression levels by qRT‐PCR analysis.

### RNA pull-down assay

RNA pull-down analysis was performed as previous report [11]. Then cytoplasmic extracts prepared from MG-63 and 143B cells using RIPA buffer were incubated with in vitro transcribed and biotinylated RNA, which were then targeted with streptavidin beads (Vector Laboratories, CA, USA) and washed. After purifified, Real-time PCR was performed to examine the expression levels of selected miRNAs.

### Animal experiments

The BALB/c nude mice (female, 4–5 weeks old) were purchased from HUAFUKANG(Beijing, China). Then the mice were randomly divided into three groups (n = 05) and the average weigh of mice was 16 g. MG-63 cells stably transfected with sh-circ_0028171 or sh-NC were subcutaneously injected into left inguinal region in mice with 10^7^ cells in 100 ul PBS. Tumor length and width were measured to calculate tumor volume according to the formula: volume (mm^3^) = (width^2^ × length)/2. Thirty days later, the mice were sacrificed and the volume and weight of their tumors were measured.

### Statistical analysis

The data are presented as the mean ± standard deviation (SD) from at least three independent experiments. Statistical significance was measured using Student’s t-test and ANOVA. The correlation was measured using Pearson’s correlation analysis. The experiments of the research in vitro were repeated at the last 3 times. GraphPad Prism 7.0 (GraphPad, La Jolla, CA, USA) and SPSS 18.0 software (SPSS, Chicago, IL, USA) were used for statistical analysis. Differences were considered significant at P < 0.05.

## Results

### circ_0028171 was highly expressed in OS tissues and cells

**Fig. 1:**
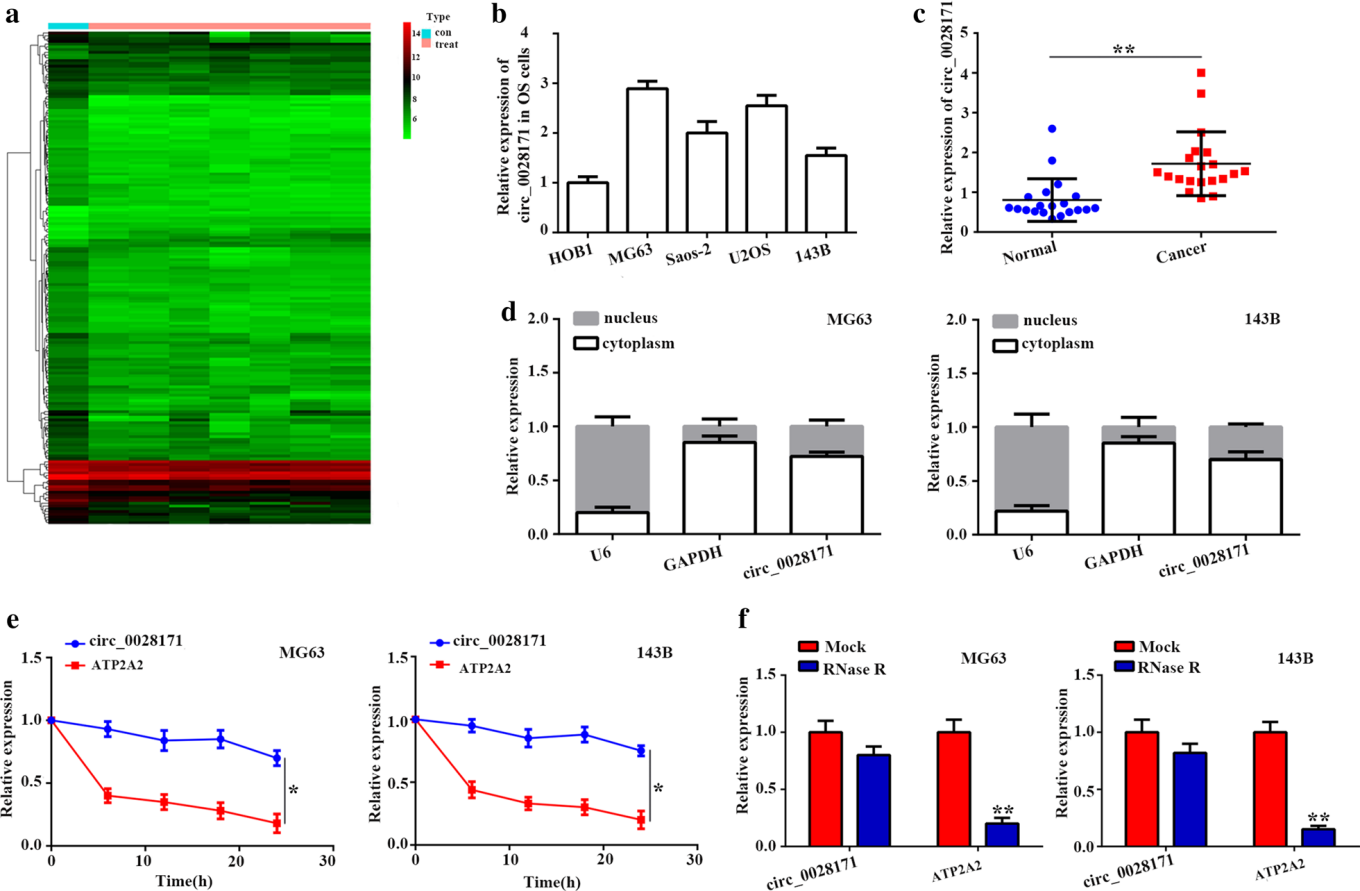
circ_0028171 was highly expressed in OS tissues and cells. **a** CircRNA microarray based on OS cell lines in GSE96964. **b** Circ-0028171 expression level in four OS cell lines relative to HOB1 cells. **c** The expression of circ_0028171 was analyzed in 20 paired tumor and normal tissues by qRT-PCR (*P < 0.01, Student’s t-test). **d** Subcellular distribution of circ_0028171was detected by qRT-PCR. **e**, **f** The expression level of circ_0028171(circ_ ATP2A2) and ATP2A2 were detected in MG-63 and 143B cells by qRT-PCR after treatment with actinomycin D and RNase R, respectively

To identify key circRNAs regulating OS development, we analyzed an online microarray dataset [31] (GSE96964) to search differentially expressed circRNAs between OS cell lines and normal osteoblast cell line. Results revealed that circ_0028171 expression was markedly higher in OS cells (Fig. 1a). Meanwhile, we verified the expression level of circ_0028171 in four kinds of OS cell lines (MG-63, Saos-2, U2OS, and 143B) was notably higher than that in HOB1 cells by qRT-PCR (Fig. 1b). qRT-PCR results again confirmed that the expression level of circ_0028171 was remarkably higher in OS tissues than in surrounding normal tissues (Fig. 1c, p < 0.01). These findings suggested that increased circ_0028171 may be critically involved in OS progression. Nuclear cytoplasmic fractionation assays showed that the expression level of circ_0028171 in cytoplasm was more abundant than that in nucler (Fig. 1d). RNA stability assay using actinomycin D and qRT-PCR quantification demonstrates that the circ_0028171 transcript half-life was more than 24 h in MG-63 and 143B cells, while most ATP2A2 mRNA had been degraded at that time (Fig. 1e). As shown in Fig. 1f, the level of ATP2A2 mRNA (a kind of linear form mRNA) was strongly decreased under the RNase R treatment, whereas circ_0028171 was more resistant to RNase R digestion. Taken together, these data indicated that circ_0028171 might be the diagnostical marker in OS patients.

### Circ_0028171 contributes to OS cell proliferation, migration and invasion

**Fig. 2:**
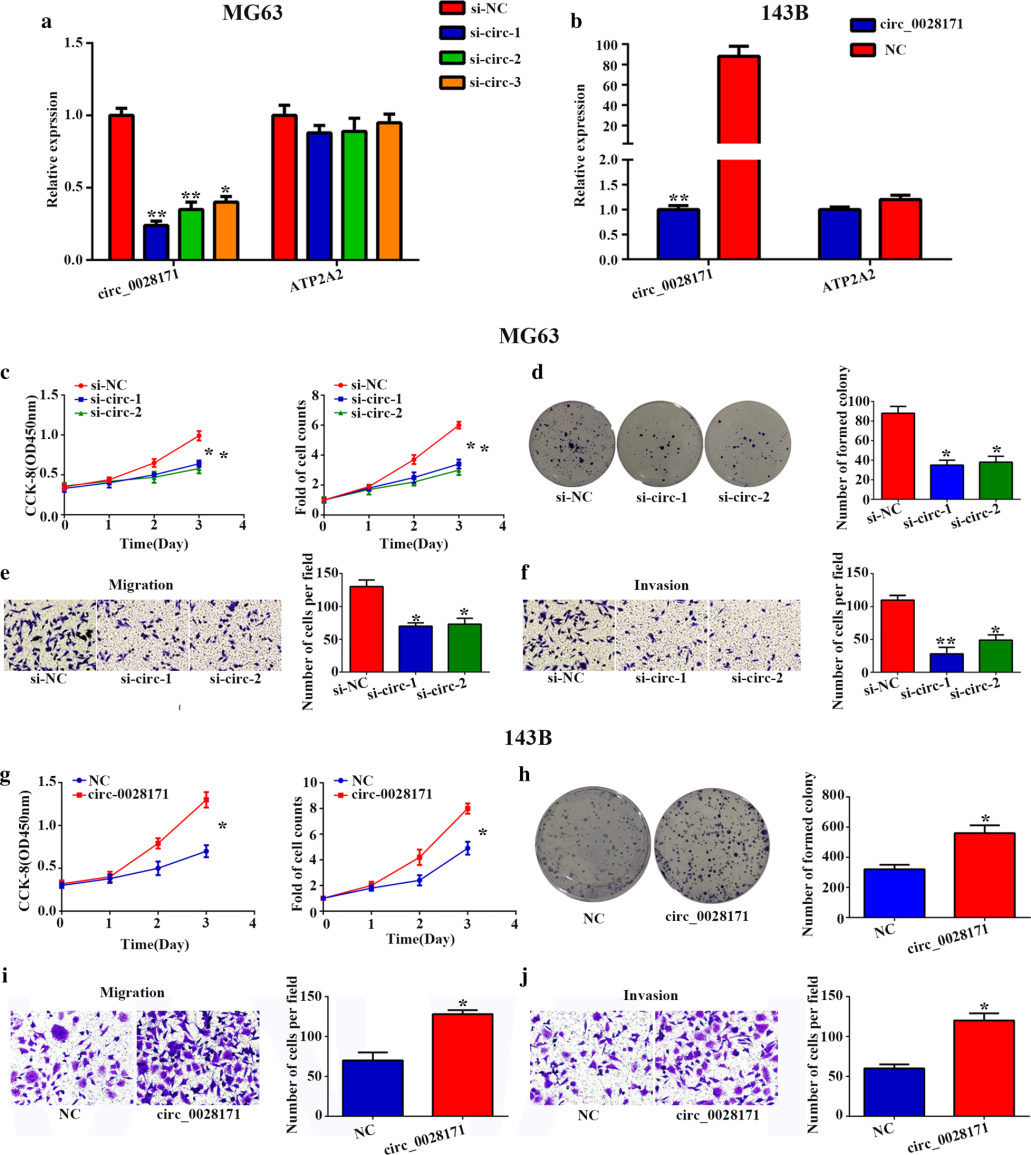
circ_0028171 promotes malignant phenotype in OS cells. **a** circ_0028171 and 0028171 expression after transfection with three siRNAs targeted circ_0028171 or control were assessed by qRT-PCR in MG-63 cells. **b** (circ_0028171 circ_ATP2A2) and ATP2A2 expression after transfection with circ_0028171 overexpressed or control plasmid were assessed by qRT-PCR in 143B cells. **c**, **d** The cell proliferation in MG-63 was examined by CCK-8, cell count assays as well as cell colony assay. **e**–**f** The transwell assays were used to assessed the migrated and invaded cells. **g**, **h** The cell proliferation in 143B cell was examined by CCK-8, cell count assays as well as cell colony assay. **i**, **j** The migrated and invaded 143B cells were evaluated by transwell assays

Subsequently, we evaluated the effect of circ_0028171 knockdown and overexpression on OS cells in vitro. qRT-PCR was conducted in MG-63 cells transfected with circ_0028171 siRNAs and 143B cells transfected with circ_0028171 to examine the knockdown and overexpressing efficiency. As shown in Fig. 2a, both si-circ-1 and si-circ-2 evidently downregulated the level of circ_0028171, but not that of 0028171 in MG-63 cells and compared with the control group (transfection with empty vector), circ-0028171 treatment caused a remarkable upregulation of circ-0028171, instead of 002871 in 143B cells (Fig. 2b). CCK-8 assays and colony formation assays displayed that depletion of circ-0028171 reduced cell viability and proliferative capacity compared with si-NC group in MG-63 cells (Fig. 2c, d). Furthermore, transwell assays showed that knockdown of circ-0028171 obviously decreased the migration and invasion of MG63 cells (Fig. 2e, f). However, circ-0028171 overexpression notably promoted cancer cell proliferation, migration and invasion in 143B cells (Fig. 2g–j). In summary, our data indicated that circ-0028171 played a crucial part in the progress of OS.

### Circ_0028171 acts as a sponge of miR-218-5p

**Fig. 3:**
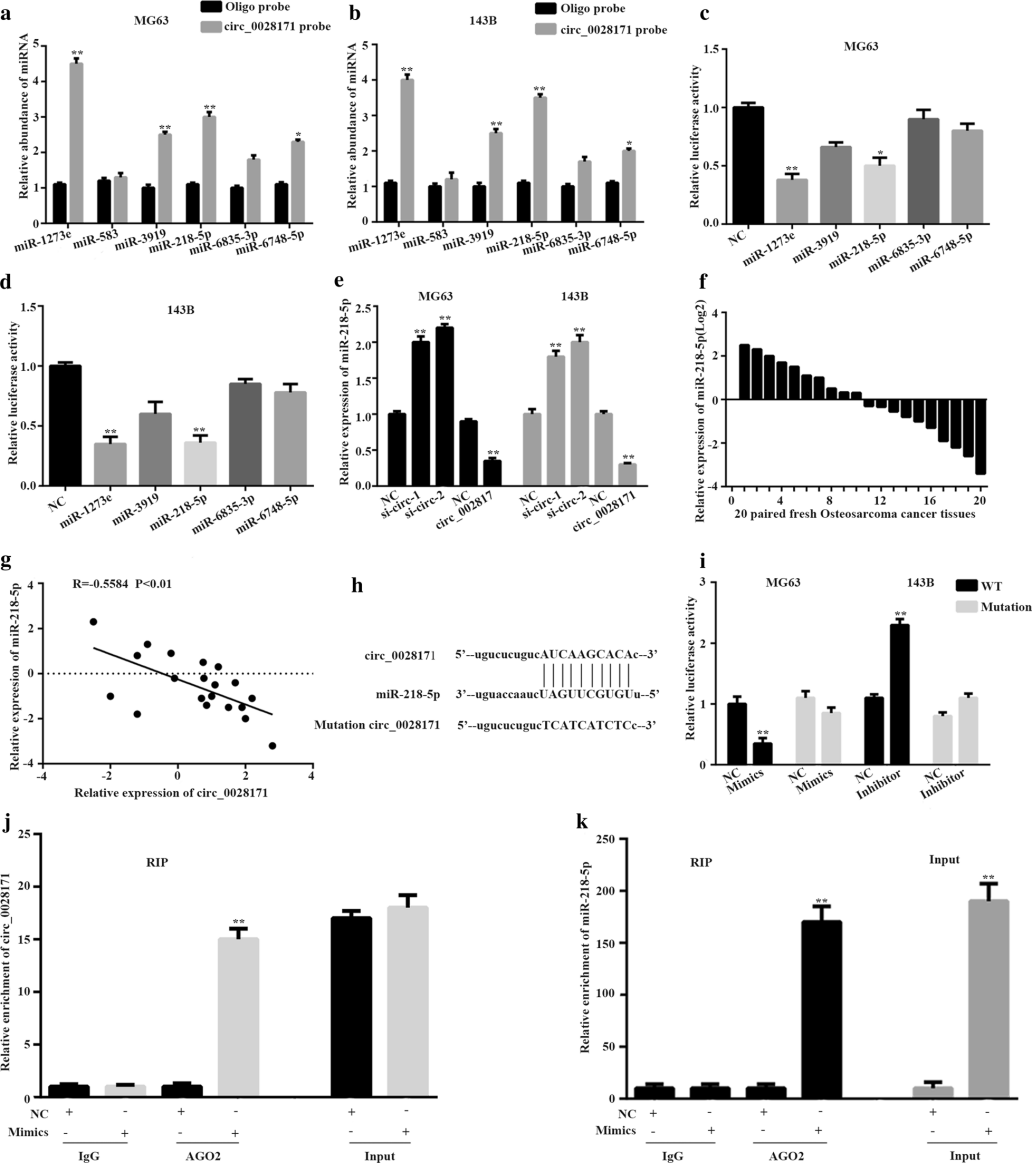
circ_0028171 acts as a sponge for miR-218-5p in osteosarcoma cells. **a**, **b** qRT-PCR was conducted to detect six miRNA candidates in MG-63 and 143B cells pulled down by circ_0028171 probe. **c**, **d** Relative luciferase intensity were detected in OS cells after co-transfecting with circ_0028171 ’UTR-wild type and miRNA mimics. **e** The change circ_0028171expression obviously influenced miR-218-5p expression in MG-63 and 143B cells. **f** miR-218-5p expression level in 20 paired OS tissues. **g** miR-218-5p and circ_0028171 expression was tested by Pearson’s correlation. **h** The miR-218-5p-binding sites in circ_0028171 sequence was predicted by bioinformation. **i** The relative luciferase activity was detected after co-transfection with circ_0028171 reporter plasmid and miR-218-5p in MG-63 and 143B cells. **j**, **k** The anti-AGO2 RIP assay was performed to detect the connection between miR-218-5p and circ_0028171

Given that circ_0028171 is mainly localized in the cytoplasm, where circRNAs serve as a miRNA to regulate genetic transcription, we guessed that hsa_circ_0028171 might exerts its function through competitive binding to downstream miRNAs. Therefore, circ_0028171 was input to predict potential miRNA regulation. We selected top 6 high confidence miRNAs for our follow-up experiments. RNA pull-down assay showed that miR-1273e, miR-3919, miR-218-5p and miR-6748-5p were captured in both MG63 and 143B cells (Fig. 3a, b). Next we performed luciferase reporter assays to confirm that miR-1273e or miR-218-5p decreased the luciferase activity of the reporter plasmid carrying the wild‐type circ_0028171(Fig. 3c, d). Furthermore, we found that miR‐218-5p expression was increased upon circ_0028171 knockdown and decreased following circ_0028171 overexpression in MG63 and 143B cells(Fig. 3e), which indicated that circ_0028171 may act as a sponge of miR‐218-5p. To confirm the expression levels of miR‐218-5p in OS patients, we detected their expression levels in above 20 OS tissues and the paired normal adjacent tissues using qRT-PCR. MiR‐218-5p was also confirmed to exhibit abnormally low expression level in most of OS tissues relative to adjacent normal tissues (Fig. 3f). Further correlation analyses found that the expression level of miR‐218-5p were negatively correlated with that of circ_0028171 in OS tissues (Fig. 3g, P < 0.01, r = − 0.5584).To determine whether circ_0028171 could directly regulate miR‐218-5p,we generated luciferase reporter constructs that contained mutated binding sites of circ_0028171 on miR‐218-5p (Fig. 3h). The subsequent Luciferase reporter assays demonstrated that the wild-type luciferase activity in MG63 cells co-transfected with miR‐218-5p mimic was significantly decreased, while it had no effect on the luciferase activity of mutant circ_0028171. However, miR‐218-5p inhibitor dramatically increased the relative luciferase activity of WT, instead of mutation (Fig. 3i). Next, we performed an RNA immunoprecipitation (RIP) assay by using an Argnonaute 2 (AGO2) antibody or control IgG. The results suggested that circ_0028171 and miR‐218-5p were significantly enriched following immunoprecipitation of AGO2 compared with IgG. Moreover, following transfection with miR‐218-5p mimics, circ_0028171 and miR‐218-5p were markedly enriched compared with negative control (Fig. 3j, k). The above results obviously illustrated that circ_0028171 is a sponge of miRNA for miR‐218-5p.

### IKBKB as the direct target gene of miR-218-5p

**Fig. 4:**
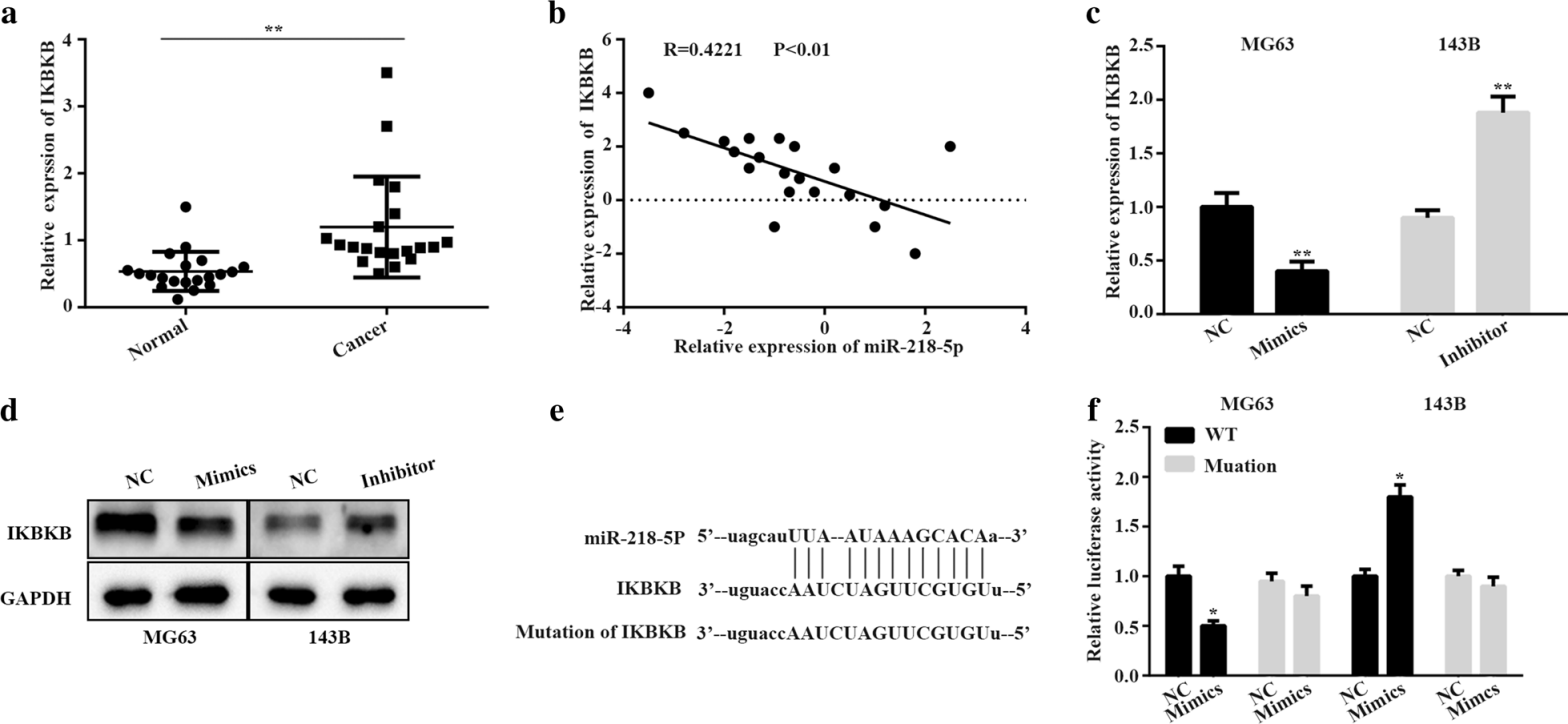
IKBKB is identified as a direct target of miR-218-5p in OS cells. **a** IKBKB expression level in 20 paired OS tissues. **b** miR-218-5p and IKBKB expression was tested by Pearson’s correlation. **c**,** d** The influence of miR-218-5p on IKBKB were analyzed by qRT-PCR and western blotting. **e** The miR-218-5p-binding sites in IKBKB sequence were predicted. **f** The relative luciferase activity was detected after co-transfection with IKBKB reporter plasmid and miR-218-5p in MG-63 and 143B cells

The target genes of miR-218-5p were predicted by four methods, including TargetScan, MicroRanda, picTAR from STARBASE (starbase.sysu.edu.cn). Among several predicted target genes, we focused on IKBKB for its high scores finally. Besides, we also found that IKBKB expression was significantly increased in above 20 paired osteosarcoma cancer tissues compared with surrounding normal tissues (Fig. 4a). Interestingly, we also found that there was an inverse correlation between the expression levels of IKBKB and miR-218-5p in OS tissues (Fig. 4b, P < 0.01, r = − 0.4221). Additionally, analysis from qRT-PCR and Western-blot also demonstrated that miR-218-5p mimics could markedly reduce the IKBKB expression in MG63cells and miR-218-5p inhibitor induced the enhancement of IKBKB expression in 143B cells (Fig. 4c, d). To examine whether miR-218-5p regulates IKBKB expression by directly binding to the predicted 3′ UTR sequence, we constructed luciferase reporter plasmids containing the wild-type or the mutated binding site for IKBKB (Fig. 4e). As expected, results of the luciferase assay revealed that WT reporter plasmid activity was potently reduced by miR-218-5p mimics in MG63cells, but it was enhanced by miR-218-5p inhibitor in 143B cells;Conversely, MUT-2 reporter plasmid activity was not obviously affected by miR-218-5p mimics or miR-218-5p inhibitor (Fig. 4f). These result support the notion that IKBKB was the target of miR-218-5p.

### Effects of IKBKB on the malignant phenotype in OS cells

**Fig. 5:**
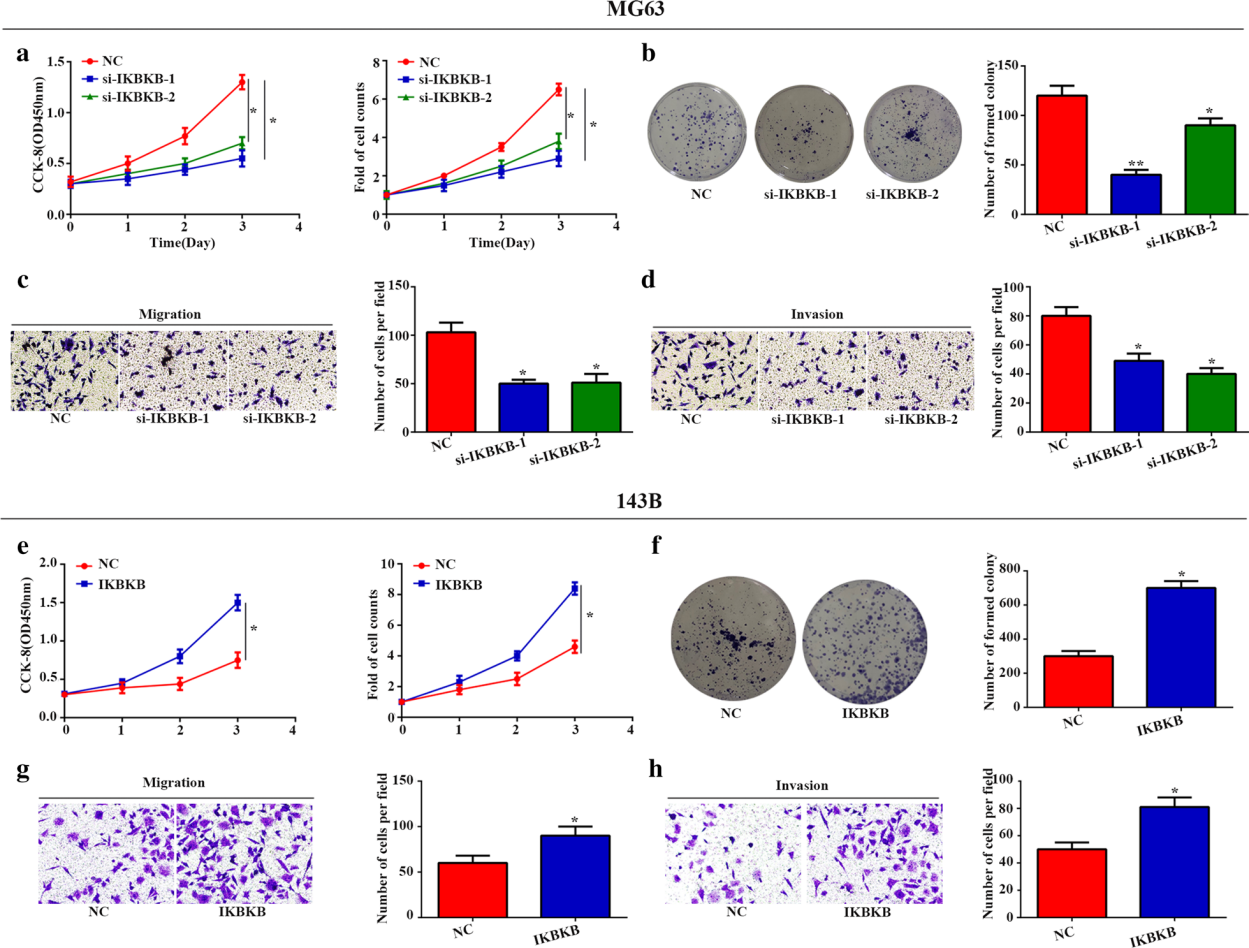
IKBKB promotes malignant phenotype in OS cells. **a** The ability of proliferation was detected by CCK-8, cell count and cell colony assays in MG-63 cells transfected with si-IKBKB. **c**, **d** The transwell assays revealed that si-IKBKB impaired cell migration and invasion of MG-63 cells. **e**, **f** The ability of proliferation was detected by CCK-8, cell count and cell colony assays in MG-63 cells transfected with IKBKB. **g**, **h** The transwell assays revealed that IKBKB accelerated cell migration and invasion of MG-63 cells

To further investigate the biological function of IKBKB in osteosarcoma, MG63 and 143B cells were transfected with the IKBKB siRNA or IKBKB-overexpressing plasmid to suppress or overexpress IKBKB expression, respectively. Knockdown of IKBKB inhibits viability and proliferative capacity compared with si-NC group in MG-63 cells, as determined by CCK-8, cell counts and colony formation assays (Fig. 5a, b). Besides down-regulation of IKBKB decreased MG-63 cells migration and invasion, as shown by the results of transwell assays (Fig. 5c, d). In contrast, we observed that the ability of viability, proliferation, migration and invasion was enhanced when IKBKB was overexpressed in 143B cells (Fig. 5e–h). Therefore, IKBKB promotes the malignant phenotype of OS cells in vitro.

### miR-218-5p influences osteosarcoma progression through IKBKB

**Fig. 6:**
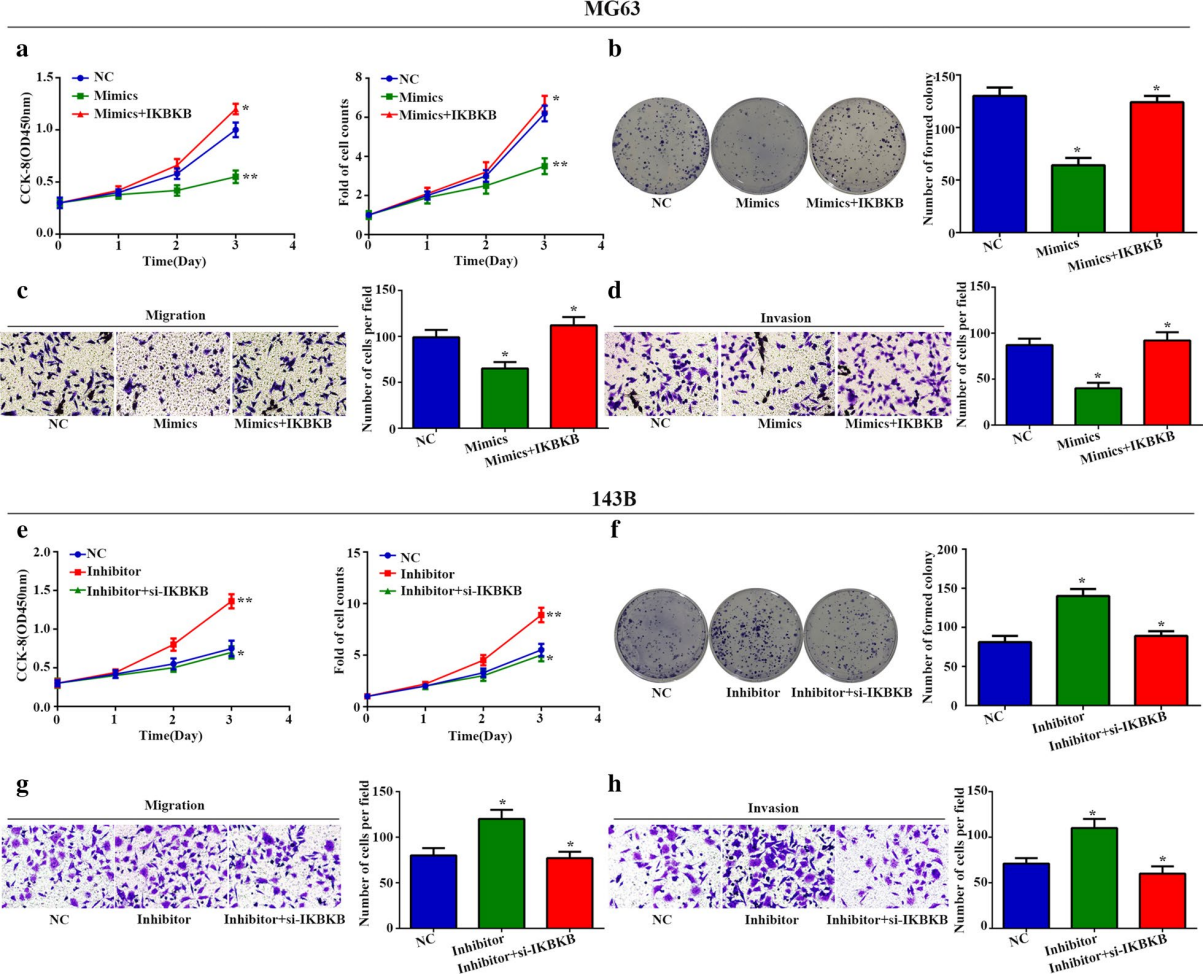
miR-218-5p suppresses malignant phenotype through IKBKB. **a**, **b** The ability of proliferation was measured by CCK-8, cell count, as well as cell colony assays. **c**, **d** The transwell assays were used to assessed the migrated and invaded cells. **e**, **f** The cell proliferation was examined by CCK-8, cell count assays as well as cell colony assay. **g**, **h** The transwell assays were used to assessed the migrated and invaded cells

Next, we explored whether miR-218-5p regulates osteosarcoma cancer progression through IKBKB. As shown in Fig. 6a–d, we found that miR-218-5p mimics could attenuate cell proliferation, migration and invasion in MG-63 cells. Moreover, we performed rescue experiments and found that the effect of miR-218-5p mimics on MG-63 cells was reversed by IKBKB overexpression. In contrast, the miR-218-5p inhibitor significantly increased the ability of viability, proliferation, migration and invasion in 143B cells, as evidenced by the results of the CCK-8 assay, cell counts, colony formation assay and transwell migration and invasion assays. consistently, silencing of IKBKB abolished the effect of miR-218-5p inhibitor on 143B cell (Fig. 6a–h). These results further confirmed that miR-218-5p inhibitor promotes osteosarcoma progression through IKBKB.

### MiR-218-5p reverses IKBKB expression and malignant phenotype caused by circ_0028171

**Fig. 7:**
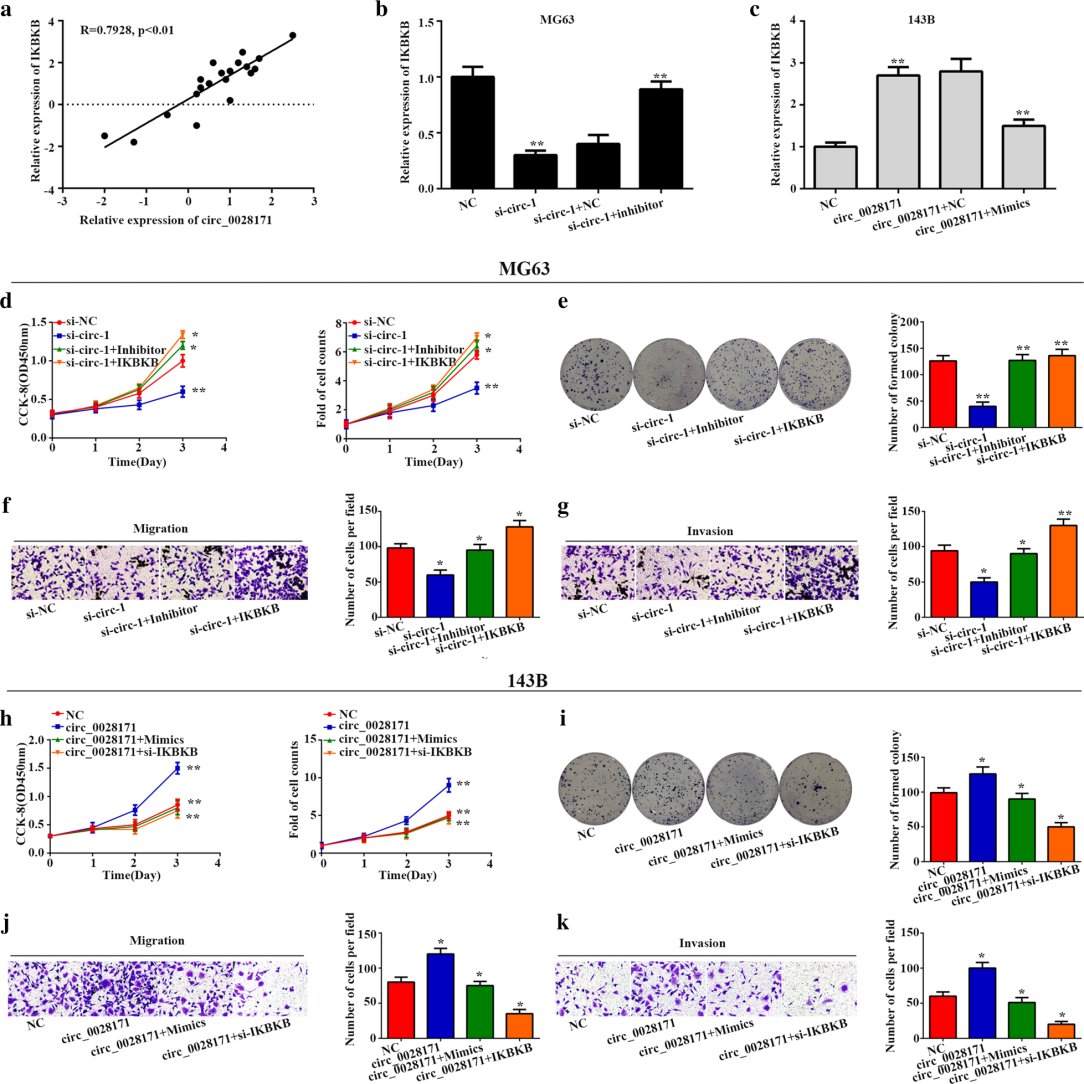
circ_0028171 facilitates OS progression through miR-218-5p and IKBKB.** a** Correlation analysis of circ_0028171 and IKBKB expression in OS tissues. **b**, **c** The impact of circ_0028171 and miR-218-5p expression on IKBKB expression. **d**–**i**) The influence of circ_0028171 and miR-218-5p or IKBKB expression on cell proliferation, migration, invasion. **h**–**k** The effects of altered circ_0028171 and miR-218-5p or IKBKB expression on the ability of proliferation, migration and invasion

Given our previous results, we found there was a positive correlation between the expression levels of IKBKB and circ_0028171 in OS tissues (Fig. 7a, P < 0.01, r = 0.7928). Then we used rescue experiments to verify the impact of MiR-218-5p on the role of circ_0028171 to IKBKB expression and malignant phenotype in osteosarcoma progression. First, we observed that miR-218-5p inhibitor efficiently restored IKBKB expression,which was repressed by si-circ_0028171 in MG-63 cells (Fig. 7b). While miR-218-5p mimics suppressed the promotion of circ_0028171 on IKBKB expression in 143B cells (Fig. 7c). Further, functional studies showed that miR-218-5p inhibitor could recover cell proliferation, migration and invasion in MG-63 cells transfected with siRNA-circ_0028171. In contrast, miR-218-5p mimics significantly attenuated the ability of circ_0028171 to promote viability, proliferation, migration and invasion in 143B cells, as showed by the results of the CCK-8 assay, cell counts, colony formation assay and transwell migration and invasion assays (Fig. 7d–k). Above all, these results illustrated that circ_0028171 took part in the progress of osteosarcoma the increasing expression of IKBKB by competing for miR-218-5p.

### The effects of circ_0028171 on tumor growth in vivo

**Fig. 8:**
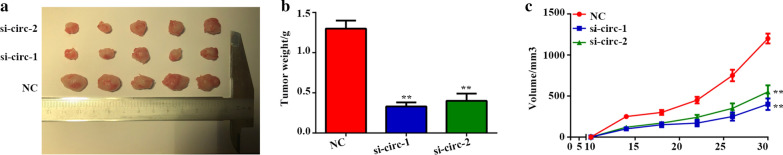
circ_0028171 promotes growth of OS in vivo. **a** tumors were dissected from in nude mice. **b** Tumor weights and **c** tumor volume of three groups

To investigate whether circ_0028171 is crucial for the progression of osteosarcoma in vivo, we established xenograft osteosarcoma models in Wistar rats to determine the effect of downregulation of circ_0028171. Mice were killed on day 28 and tissue samples harvested (Fig. 8a). As Fig. 8b, c indicated, the tumor volume and tumor weight were obviously lower in si-circ-1and si-circ-2 group compared with NC group. Therefore, we speculate that down-regulation of circ_0028171 exerts a therapeutic effect in a rat model of osteosarcoma.

## Discussion

More and more studies [32, 33] demonstrated that circRNAs participated in cancer pathogenesis by regulating mutiple biological processes, such as proliferation, migration, invasion. For instance, Lu et al. [34] reported that the expression level of circ_0008792 was low in OS cell lines, while restored the expression of circ_0008792 could inhibit malignant phenotypes in OS, such as cell migration and invasion.. Zhang et al. [35] showed that circCCDC66 was overexpressed in CDDP-resistant GC and couod serve as a biomarker for poor prognosis. In the present study, we first analyzed an online microarray dataset (GSE96964) and found that circ_0028171 expression was significantly up-regulated in OS cell lines. Then, qRT-PCR was performed to detect the level of circ_0028171 in 20 paired OS tissues and adjacent normal tissues to further confirmed our previous results. We additionally explored the function of circ_0028171 through CCK-8, cell count, cell colony and transwell assys, and found that knockdown circ_0028171 remarkably attenuate the proliferation, migration and invasion abilities in OS cells. On the contrary, we observed the opposite result when circ_0028171 was overexpressed, indicating that circ_0028171 was crucial to OS progression.

Over the past few years, miRNA sponge effect of circRNAs located in the cytoplasm was the most common and intensively studied mechanism [36]. For example, circCLK3 [11] acted as a miRNA sponge to decrease expression of miR-320a, which targeted and repressed FoxM1 expression, and thereby promotes a variety of malignant phenotypes of cervical cancer, such as cell proliferation, EMT, migration and invasion. Intrestingly, our analysis revealed that the expression level of miR-218-5p were negatively correlated with that of circ_0028171 in OS tissues. Luciferase reporter assays discovered that circ_0028171 contains miR-218-5p response elements and binding sites. Furthermore, we found that miR-218-5p minics significantly attenuated the ability of circ_0028171 to promote viability, proliferation, migration and invasion, suggesting that miR-218-5p served as a tumor suppressor in OS. The above results supported the conclusion that circ_0028171 is a sponge of miRNA for miR-218-5p.

In the last few years, many studies [25, 26, 37] had confirmed that microRNA (miRNA or miR) regulates gene expression by directly binding to 3′-untranslated regions of target mRNA. Based on our experimental results, IKBKB was one of the target gene of miR-218-5p and could be repressed by miR-218-5p minic, which to our knowledge had not been reported so far. In addition, our present study revealed that IKBKB expression was significantly upregulated in above 20 paired osteosarcoma cancer tissues compared with adjacent normal tissues. According to previous research reports [28, 38], the IKBKB was involved in multiple cellular processes, including inflammation and immunity. Lim et al. [39] had reported that NF-κB signalling pathway activated by IKBKB could promote the expression of PD-L1 at the transcriptional as well as the post-transcriptional level. As we all know, PD-1 protein can bind to its cognate receptor, PD-1 antibody, on T cells, which results in the suppression of the immune response. [40]. Our results showed that IKBKB promotes viability, proliferation, migration and invasion of OS cells in vitro. These results corroborated the findings of a great deal of the previous work in the biological function of IKBKB of regulating tumor inflammatory microenvironment. From all the above experiments, we demonstrated that circ_0028171 promoted the malignant progress of OS cells via regulating IKBKB expression via sponging miR-218-5p. Additionally, in the current study, we only explored the function and mechanism of circ_0028171/miR-218-5p/IKBKB axis in OS. However, the detail regulation mechanism about circ_0028171 and why the circ_0028171 is upregulation in the OS were still unclear. In the future, the regulation of circ_0028171 overexpressed and some signaling pathways involved in the mechanism of circ_0028171/miR-218-5p/IKBKB axis can be studied in depth.

## Conclusions

In conclusion, we have confirmed that the expression level of circ_0028171 was significantly up-regulated in OS cell lines and OS tissues and we first demonstrated that circ_0028171 contributes to the ability of proliferation, migration and invasion of OS cells in vitro by sponging miR-218-5p to improve the expression of IKBKB. These results suggest that circ_0028171 may be a potential novel biomarker for diagnosis and treatment target of OS.

## Data Availability

All data generated or analyzed during this study are included in this published article.
